# Parental genetically predicted liability for coronary heart disease and risk of adverse pregnancy outcomes: a cohort study

**DOI:** 10.1186/s12916-023-03223-9

**Published:** 2024-01-25

**Authors:** Álvaro Hernáez, Karoline H. Skåra, Christian M. Page, Vera R. Mitter, Marta H. Hernández, Per Magnus, Pål R. Njølstad, Ole A. Andreassen, Elizabeth C. Corfield, Alexandra Havdahl, Øyvind Næss, Ben Brumpton, Bjørn Olav Åsvold, Deborah A. Lawlor, Abigail Fraser, Maria Christine Magnus

**Affiliations:** 1 Centre for Fertility and Health, Norwegian Institute of Public Health, Skøyen, 0213, PO 222, Oslo, Norway; 2 Blanquerna School of Health Sciences, Universitat Ramon Llull, Barcelona, Spain; 3 Centro de Investigación Biomédica en Red de Enfermedades Cardiovasculares (CIBERCV), Instituto de Salud Carlos III, Madrid, Spain; 4 Department of Community Medicine and Global Health, Institute of Health and Society, University of Oslo, Oslo, Norway; 5 Department of Physical Health and Ageing, Division for Mental and Physical Health, Norwegian Institute of Public Health, Oslo, Norway; 6 Pharmacoepidemiology and Drug Safety Research Group, Department of Pharmacy, and PharmaTox Strategic Research Initiative, Faculty of Mathematics and Natural Sciences, University of Oslo, Oslo, Norway; 7 Center for Diabetes Research, Department of Clinical Science, University of Bergen, Bergen, Norway; 8 Children and Youth Clinic, Haukeland University Hospital, Bergen, Norway; 9 Norwegian Centre for Mental Disorders Research, NORMENT, Division of Mental Health and Addiction, Oslo University Hospital, Oslo, Norway; 10 Institute of Clinical Medicine, University of Oslo, Oslo, Norway; 11 Center for Genetic Epidemiology and Mental Health, Norwegian Institute of Public Health, Oslo, Norway; 12 Nic Waals Institute, Lovisenberg Diakonale Hospital, Oslo, Norway; 13 ROMENTA Research Center, Department of Psychology, P, University of Oslo, Oslo, Norway; 14 Population Health Sciences, Bristol Medical School, University of Bristol, Bristol, UK; 15 Institute of Health and Society, University of Oslo, Oslo, Norway; 16 Division of Mental and Physical Health, Norwegian Institute of Public Health, Oslo, Norway; 17 K.G. Jebsen Center for Genetic Epidemiology, Department of Public Health and Nursing, Norwegian University of Science and Technology, Trondheim, Norway; 18 HUNT Research Centre, Department of Public Health and Nursing, Norwegian University of Science and Technology, Levanger, Norway; 19 Clinic of Medicine, St. Olavs Hospital, Trondheim University Hospital, Trondheim, Norway; 20 Department of Endocrinology, Clinic of Medicine, St. Olavs Hospital, Trondheim University Hospital, Trondheim, Norway; 21 MRC Integrative Epidemiology Unit, University of Bristol, Bristol, UK

**Keywords:** Coronary heart disease, Adverse pregnancy outcomes, Parental genetic liability to CHD, MoBa, HUNT

## Abstract

**Background:**

Adverse pregnancy outcomes (APO) may unmask or exacerbate a woman’s underlying risk for coronary heart disease (CHD). We estimated associations of maternal and paternal genetically predicted liability for CHD with lifelong risk of APOs. We hypothesized that associations would be found for women, but not their male partners (negative controls).

**Methods:**

We studied up to 83,969‬ women (and up to 55,568‬ male partners) from the Norwegian Mother, Father and Child Cohort Study or the Trøndelag Health Study with genotyping data and lifetime history of any APO in their pregnancies (1967–2019) in the Medical Birth Registry of Norway (miscarriage, stillbirth, hypertensive disorders of pregnancy, gestational diabetes, small for gestational age, large for gestational age, and spontaneous preterm birth). Maternal and paternal genetic risk scores (GRS) for CHD were generated using 148 gene variants (*p*-value < 5 × 10^−8^, not in linkage disequilibrium). Associations between GRS for CHD and each APO were determined using logistic regression, adjusting for genomic principal components, in each cohort separately, and combined using fixed effects meta-analysis.

**Results:**

One standard deviation higher GRS for CHD in women was related to increased risk of any hypertensive disorders of pregnancy (odds ratio [OR] 1.08, 95% confidence interval [CI] 1.05–1.10), pre-eclampsia (OR 1.08, 95% CI 1.05–1.11), and small for gestational age (OR 1.04, 95% CI 1.01–1.06). Imprecise associations with lower odds of large for gestational age (OR 0.98, 95% CI 0.96–1.00) and higher odds of stillbirth (OR 1.04, 95% CI 0.98–1.11) were suggested. These findings remained consistent after adjusting for number of total pregnancies and the male partners’ GRS and restricting analyses to stable couples. Associations for other APOs were close to the null. There was weak evidence of an association of paternal genetically predicted liability for CHD with spontaneous preterm birth in female partners (OR 1.02, 95% CI 0.99–1.05), but not with other APOs.

**Conclusions:**

Hypertensive disorders of pregnancy, small for gestational age, and stillbirth may unmask women with a genetically predicted propensity for CHD. The association of paternal genetically predicted CHD risk with spontaneous preterm birth in female partners needs further exploration.

**Supplementary Information:**

The online version contains supplementary material available at 10.1186/s12916-023-03223-9.

## Background

Adverse pregnancy outcomes (APO), such as miscarriage, stillbirth, hypertensive disorders of pregnancy (HDP), gestational diabetes (GD), small for gestational age (SGA), large for gestational age (LGA), and spontaneous preterm birth (sPTB), may unmask a woman’s underlying liability for cardiovascular disease, identifying women who “fail” the cardiometabolic stress test of pregnancy [1]. Preconception cardiovascular risk factors are associated with APOs [2–7] and the extent of physiological changes of pregnancy including vasodilation, decreases in glucose, and changes in lipoprotein subclasses and biomarkers of low-grade inflammation [8]. Moreover, adjustment for preconception cardiovascular risk factors attenuates the relationship between APOs and maternal post-partum risk of cardiovascular disease [9–11]. An association between genetically predicted liability for cardiovascular disease and APOs in women would offer further support to the “unmasking” hypothesis as germline variants are inherited at birth. These analyses would also provide additional evidence on alternative hypothesis to explain the association, such as the possible direct role of APOs (such as HDP) on accentuating the risk of developing cardiovascular diseases in the future [12, 13].

We therefore investigated associations between maternal and paternal genetic liability for coronary heart disease (CHD) with APOs (miscarriage, stillbirth, HDP, GD, SGA, LGA, and sPTB). We hypothesized that we would find an association in women but not in men, as men’s genetic predisposition to CHD would not be revealed when their female partner undergoes the cardiometabolic stress test of pregnancy. Hence, we considered men as imperfect negative controls. Imperfect, because shared family environment associated with both genetic liability for CHD and APO risk (e.g., body mass index, smoking) [14] may result in associations between genetic liability for CHD in men and APO risk in their female partners. Paternal genetic variants linked to CHD might also impact the epigenetics of their reproductive cells or the quality of their sperm, and these factors may in turn influence APO risk [15, 16]. Finally, fetal genetics inherited from both parents may also affect risk of APOs [17, 18]. We used genetically predicted CHD risk as the exposure because CHD is the most common cardiovascular disease, the leading cause of death globally, and the cardiovascular event with the highest proportion of genetic variance explained in genome-wide association studies (GWAS) [19].

## Methods

### Population description

We studied participants in the Norwegian Mother, Father, and Child Cohort Study (MoBa) [20] and the Trøndelag Health Study (HUNT) [21]. MoBa is a pregnancy cohort study led by the Norwegian Institute of Public Health in which pregnant women and their partners were recruited at approximately 17 gestational weeks between 1999 and 2008 all over Norway. The participation rate was 41%, and the cohort includes approximately 95,200 women and 75,200 of their male partners [20]. The HUNT study is a population-based cohort of the Trøndelag County in Norway (representative of the general adult Norwegian population regarding morbidity, mortality, income, and age), led by the Norwegian University of Science and Technology and based on four data collection surveys between 1984 and 2019 [21, 22]. Both MoBa and HUNT participants are of predominantly European ancestry. Information from the Medical Birth Registry of Norway (MBRN) for participants in both cohorts was obtained by linkage using unique identification numbers.

**Fig. 1:**
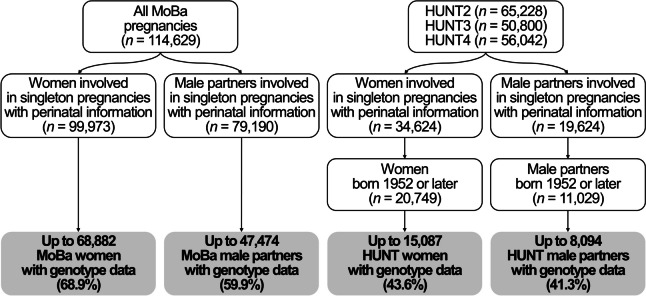
Flow chart

This work is based on a subsample of participants in the two cohorts who had at least one registered singleton pregnancy in the MBRN, available genotype data, and information on APOs (Fig. 1). Regarding HUNT, as the MBRN has information on all births in Norway from 1967 onwards [23], we restricted our analyses to cohort participants who were 15 years or younger at the time when the MBRN was set up (born 1952 or later), to capture all of their births. Genotype data in both cohorts came from biological samples obtained from participants after genotype calling, imputation, and quality control [22, 24]. This work is presented according to the Strengthening the Reporting of Observational Studies in Epidemiology guidelines.

### Genetic liability for coronary heart disease

We obtained publicly available summary data on the genetic variants linked to CHD from the latest GWAS including ~ 550,000 individuals from the UK Biobank and CARDIoGRAMplusC4D cohorts [19]. No MoBa or HUNT participants took part in this GWAS. It identified 148 genetic variants (single-nuclear polymorphisms) associated with CHD at genome-wide significance (*p*-value < 5 × 10^−8^) and independent of each other (i.e., not in linkage disequilibrium [pairwise *r*^2^ < 0.1 and a physical distance of at least 5000bps]) [25]. One hundred forty-one and 146 of these genetic variants were available in the MoBa and HUNT databases, respectively. We used these variants to calculate weighted genetic risk scores (GRSs) for CHD, representing the genetic liability for CHD [26]. The exact genetic variants used in each cohort is available in Additional file 1: Table S1.

### Adverse pregnancy outcomes

APOs were defined using information from the MBRN for both cohorts. We identified individuals in MoBa and HUNT with any history of any APO across all their registered pregnancies from 1967 to 2019. Miscarriage was defined as any fetal loss prior to 23 completed gestational weeks, while stillbirth was defined as any fetal death after 23 completed gestational weeks, as registered in the MBRN [23]. The MBRN contains self-reported information on the number of prior miscarriages and stillbirths at the time of each registered pregnancy (it is not possible to distinguish the exact gestational age of prior pregnancies not registered in the MBRN), in addition to the status and gestational age of the registered pregnancies. We combined the self-reported information on previous pregnancies and the registered pregnancies to define complete history of miscarriage and stillbirth. HDP was defined as having any registration of gestational hypertension, preeclampsia, eclampsia, or hemolysis, elevated liver enzymes and low platelets syndrome in the absence of hypertension prior to the pregnancy [27]. Registrations of GD in the MBRN are made according to the criteria of the Norwegian Society of Gynecology and Obstetrics in the absence of a history of diabetes [28]. Notably, there is no information on blood pressure measurements, proteinuria levels, or glucose levels in the MBRN. The registrations of HDP and GD are therefore made based on the national clinical guidelines at the time [27, 28]. Gestational age was estimated using ultrasound around 18 completed gestational week or last menstrual period for the small proportion without an ultrasound assessment. SGA was defined as birth weight < 10th percentile for gestational age and biological sex [29], while LGA was defined as birth weight ≥ 90th percentile for gestational age and biological sex of the offspring. Participants who delivered an SGA baby were excluded from the analyses on LGA and vice versa. Any non-medically induced delivery prior to the 37th week of pregnancy was considered a sPTB (induced deliveries and C-sections were excluded from the reference group) [30].

### Descriptive variables

To describe the characteristics of our population, we obtained information on birth year (value in the first pregnancy in MBRN, continuous), highest obtained parity according to MBRN (continuous), whether the study participants were involved in pregnancies with more than one partner (yes/no), years of education (value registered in the first MoBa/HUNT questionnaire available, continuous), body mass index (value registered in the first MoBa/HUNT questionnaire available, in kg/m^2^), having ever smoked (yes/no), and highest obtained parity (stillbirths plus live births according to the MBRN, continuous) [31, 32].

### Statistical analyses

We describe the distributions of normally distributed continuous variables using means and standard deviations (SDs), non-normally distributed continuous variables using medians and 1st–3rd quartiles, and categorical variables using numbers and percentages. We assessed differences between participants with and without genotype information using *t*-tests (normally distributed continuous variables), Mann–Whitney *U*-tests (non-normally distributed continuous variables), and chi-squared tests (categorical variables).

Using logistic regression, we studied the association of one SD higher maternal GRS for CHD with lifelong risk of APOs, and the association of one SD higher paternal GRS for CHD with lifelong risk of APOs in their female partners (as imperfect negative controls). We considered each woman’s reproductive history as a singular observational unit (we evaluated whether a woman or female partner experienced any APOs over her lifetime). We analyzed MoBa and HUNT data individually, and subsequently meta-analyzed their estimates using a fixed effects model, assuming participants from both cohorts come from the same underlying population and any difference in associations is random. We tested this assumption using the Cochrane Q-test for between-study heterogeneity, using a *p*-value threshold of < 0.1 as an indication of heterogeneity given the statistical inefficiency of between-study heterogeneity tests and the smaller sample size of HUNT compared to MoBa. If significant heterogeneity was detected for any of the outcomes, we meta-analyzed the estimates of the two studies using a random effects model as a complementary analysis. We reduced confounding of the relationship between GRSs for CHD and the outcomes due to population stratification by adjusting our analyses for the first 20 ancestry-informative principal components [33]. Analyses were further adjusted for genotype batch.

As parental negative control analyses (i.e., comparing maternal associations to paternal associations) can be biased by assortative mating and other shared environmental determinants if they are not mutually adjusted for each other [34], we repeated our main analyses in the subgroup of mothers and their partners where both had genetic data. We present results in this subgroup without and with mutual adjustment. The former analysis, when compared with the main results, explores evidence of selection bias in the subsample in couples with genetic data.

We conducted additional sensitivity analyses. Firstly, the more pregnancies a person has, the higher the likelihood of detecting an effect of parental genetic predisposition to CHD on the risk of APO. To minimize this bias, we further adjusted for the total number of pregnancies the participant had. Secondly, a participant who had pregnancies with various partners might have a differing risk of APO with each partner. Therefore, we performed another sensitivity analysis that was confined to stable couples only (i.e., participants without pregnancies with different partners according to the MBRN).

#### Software

We conducted our analyses in R Software v. 4.0.3. Our analysis code is available in https://github.com/alvarohernaez/GRS_CHD_pregnancy_complications_MoBa_HUNT/.

## Results

### Study population

Meta-analyses of the main results were conducted across different subsets of women and men. Regarding women: 75,210 for miscarriage, 83,900 for stillbirth, 83,114 for HDP, 83,900 for GD, 66,110 for SGA, 69,789 for LGA, and 83,522 for sPTB. Regarding men: 51,156 for miscarriage, 55,790 for stillbirth, 55,273 for HDP, 55,790 for GD, 43,967 for SGA, 46,104 for LGA, and 55,536 for sPTB. Characteristics of MoBa and HUNT participants involved in the meta-analyses are described in Table 1.

**Table 1:** Population description

	MoBa		HUNT	
	Women (*n* = 68,882)	Male partners (*n* = 47,474)	Women (*n* = 15,087)	Male partners (*n* = 8,094)
Birth year, range	1954–1993	1938–1990	1952–1988	1952–1988
Highest obtained parity (1st–3rd quartile; range)	2 (2–3) [range: 1–14]	2 (2–3) [range: 1–14]	2 (2–3) [range: 1–10]	2 (2–3) [range: 1–10]
Participants in pregnancies with more than one different partner, *n* (%)	9147 (13.3%)	5819 (12.3%)	745 (4.74%)	178 (2.08%)
Age in first pregnancy, median (1st–3rd quartile)	27 (24–30)	29 (26–33)	24 (20–27)	26 (23–29)
Education years, mean ± SD	17.0 ± 3.36	16.3 ± 3.56	14.3 ± 4.13	13.5 ± 4.08
Body mass index (kg/m^2^), median (1st–3rd quartile)	23.1 (21.1–25.9)	25.4 (23.6–27.7)	25.2 (22.7–28.6)	26.6 (24.5–29.1)
Ever smokers, *n* (%)	35,631 (52.3%)	24,294 (51.2%)	8,865 (57.0%)	4,233 (50.1%)
Total number of deliveries:				
0, *n* (%)	0 (0%)	0 (0%)	1,598 (10.5%)	595 (7.00%)
1, *n* (%)	4,611 (6.71%)	2,894 (6.12%)	6,849 (45.1%)	4,041 (47.5%)
2, *n* (%)	32,186 (46.8%)	23,368 (49.4%)	5,088 (33.5%)	3,056 (36.0%)
3, *n* (%)	24,121 (35.1%)	16,357 (34.6%)	1,285 (8.46%)	654 (7.69%)
4 or more, *n* (%)	7,784 (11.3%)	4,671 (9.88%)	378 (2.49%)	154 (1.81%)
Miscarriage, *n* (%)	20,866 (30.4%)	14,451 (30.6%)^b^	1,950 (30.0%)^a^	1,145 (29.6%)^a,b^
Stillbirth, *n* (%)	872 (1.27%)	581 (1.23%)^b^	198 (1.30%)	84 (0.99%)^b^
Hypertensive disorders of pregnancy, *n* (%)	7,288 (10.6%)	5,186 (11.0%)^b^	1,500 (9.87%)	870 (10.2%)^b^
Preeclampsia + eclampsia, *n* (%)	5,143 (7.49%)	3,589 (7.59%)^b^	1,080 (7.11%)	641 (7.54%)^b^
Gestational diabetes, *n* (%)	1,495 (2.18%)	1,041 (2.20%)^b^	127 (0.84%)	62 (0.73%)^b^
Small for gestational age, *n* (%)	10,552 (15.8%)	7,354 (16.0%)^b^	1,544 (10.2%)	862 (10.1%)^b^
Large for gestational age, *n* (%)	14,620 (21.7%)	9,880 (21.3%)^b^	1,733 (11.4%)	1,070 (12.6%)^b^
Spontaneous preterm birth, *n* (%)	6,282 (9.18%)	4,187 (8.89%)^b^	2,643 (17.5%)	1,509 (17.8%)^b^

^a^Analyses restricted to 1998 or later pregnancies

^b^Adverse pregnancy outcomes in their female partners

MoBa participants with genotype information were not meaningfully different in their birth year, total number of pregnancies, proportion of stable couples, age at first pregnancy, years of education, body mass index, smoking, or number of deliveries in relation to those without genotype data. However, they were slightly less likely to have experienced stillbirth, GD, SGA, and sPTB (Additional file 1: Table S2). HUNT participants with genotype data were born on average 12 years earlier but were not meaningfully different on their total number of pregnancies, proportion of stable couples, age at first birth, years of education, body mass index, and smoking. However, they were more like to experience several APOs (stillbirth, HDP, SGA, LGA, and sPTB) and less likely to have a history of GD (Additional file 1: Table S2).

### Genetically predicted CHD and adverse pregnancy outcomes

**Fig. 2:**
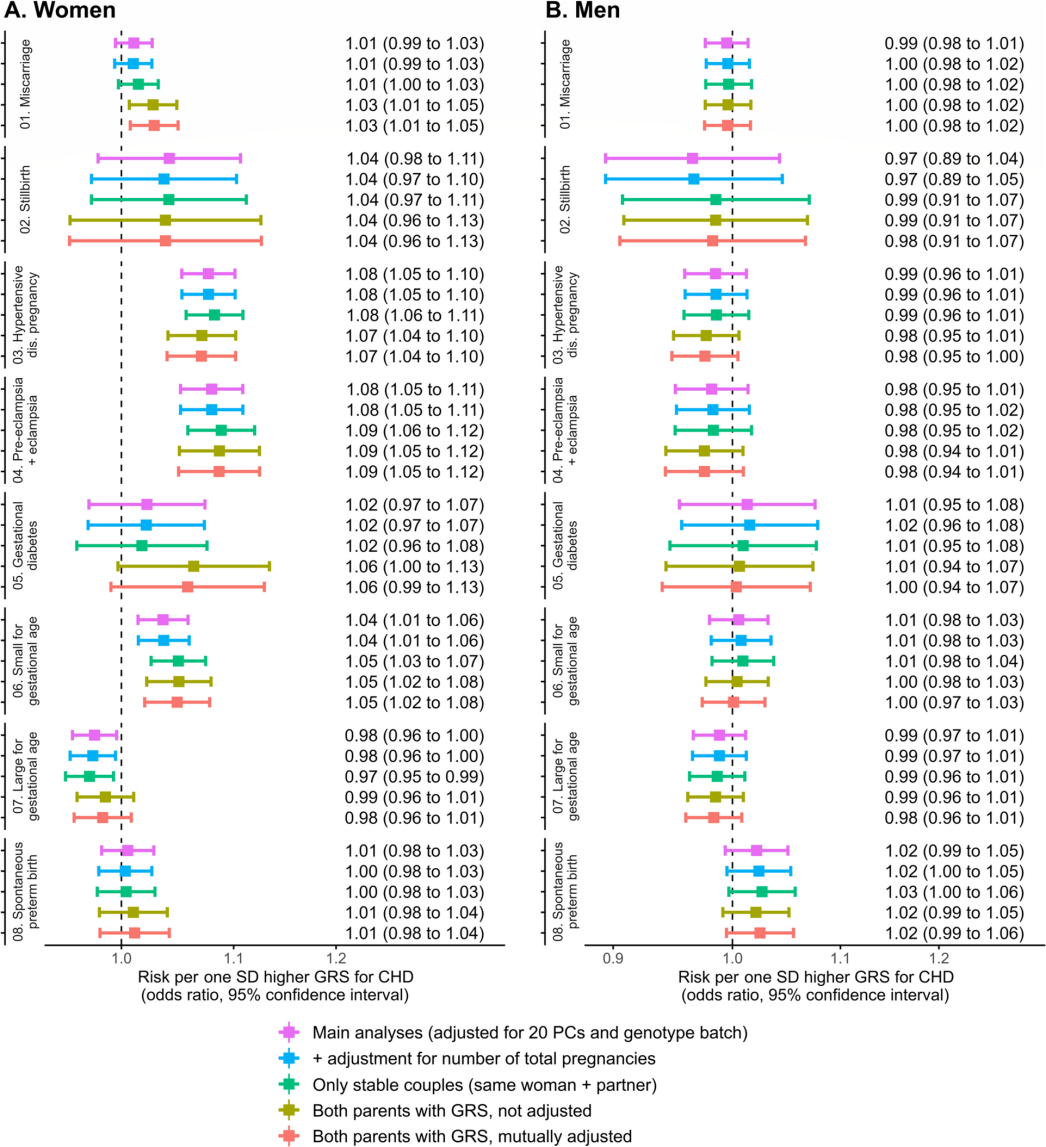
Associations between one SD higher maternal GRS for CHD and risk of APO and between one SD higher paternal GRS and risk of APO in female partners. Main analyses (pooled analyses from MoBa and HUNT participants) were conducted in 75,210 women for miscarriage, 83,900 for stillbirth, 83,114 for HDP, 83,900 for GD, 66,110 for SGA, 69,789 for LGA, and 83,522 for sPTB. Regarding men, APOs were detected in their female partners, and analyses were conducted in 51,156 individuals for miscarriage, 55,790 for stillbirth, 55,273 for HDP, 55,790 for GD, 43,967 for SGA, 46,104 for LGA, and 55,536 for sPTB. Sample sizes of additional analyses are described in Supplementary Table 3, being smallest for the analyses in the subgroup with genetic data on both parents

Figure 2 shows the main analyses and all additional and sensitivity analyses for the association of maternal GRS with risk of APOs (Panel A) and the association of paternal GRS with risk of APOs in their female partners (Panel B).

In women, one SD higher GRS for CHD was associated with 8% greater odds of HDP (OR 1.08, 95% CI 1.05 to 1.10), 8% greater odds of preeclampsia (OR 1.08, 95% CI 1.05 to 1.11), and 4% greater odds of having an SGA baby (OR 1.04, 95% CI 1.01 to 1.06). There was evidence of an inverse association between the GRS for CHD and LGA (+ 1 SD in GRS: OR 0.98, 95% CI 0.96 to 1.00). A positive but imprecise relationship was found for stillbirth (OR 1.04, 95% CI 0.98 to 1.11) and no association was found with sPTB. These findings remained consistent in the subset with genetic data on both parents when adjusting for partners’ GRS, as well as in sensitivity analyses adjusting for the number of total pregnancies and restricting the analyses to stable couples. Stronger associations between the GRS for CHD and the risk of miscarriage and GD were observed in couples with genotype information in both parents.

No association between the paternal GRS for CHD and any APO in female partners was observed, except for evidence of a relationship with greater odds of sPTB (OR 1.02, 95% CI 0.99 to 1.05). Estimates remained robust in all sensitivity analyses.

Between-study heterogeneity was detected for two outcomes: the association of the maternal GRS for CHD with GD (MoBa: OR 1.04, 95% CI 0.99 to 1.09; HUNT: OR 0.86, 95% CI 0.72 to 1.03; *p*-value for between-study heterogeneity = 0.045; random effects meta-analysis: OR 0.96, 95% CI 0.30 to 3.09), and the association of the paternal GRS with sPTB in female partners (MoBa: OR 1.04, 95% CI 1.01 to 1.07; HUNT: OR 0.97, 95% CI 0.92 to 1.03; *p*-value for between-study heterogeneity = 0.053; random effects meta-analysis: OR 1.01, 95% CI 0.68 to 1.50). For the rest of the outcomes, findings were consistent between the two cohorts (Additional file 1: Fig. S1). Sample sizes for additional analyses are available in Additional file 1: Table S3.

## Discussion

Maternal genetic CHD risk was associated with increased risk of HDP, preeclampsia, and SGA. Weak evidence with confidence intervals spanning the null was found for associations with greater risk of stillbirth and reduced risk of LGA. Null associations with miscarriage, GD, and sPTB were observed. We also found weak evidence of an association of paternal genetic CHD risk with sPTB in female partners, with associations with other outcomes all being close to the null.

Our findings support the hypothesis that a woman’s genetic predisposition to cardiovascular disease may be revealed during pregnancy or exacerbated by APOs, specifically through HDP and SGA [1]. The lack of association between male partners’ GRS for CHD and these conditions in their female partners supports these hypotheses. This agrees with previous evidence showing that women who developed these APOs had a greater burden of cardiovascular risk factors before pregnancy [5, 6]. Moreover, high levels of genetically determined cardiovascular risk factors, such as blood pressure, body mass index, and type 2 diabetes, have been linked to a higher likelihood of preeclampsia or eclampsia [35]. Finally, a recent maternal GWAS of preeclampsia and gestational hypertension found similar associations between the preeclampsia and gestational hypertension polygenic risk scores and hypertension and related outcomes as those seen in GWASs of men and non-pregnant women, leading authors to conclude that the loci associated with preeclampsia and gestational hypertension were not pregnancy specific, rather that pregnancy unmasks existing risk [13].

In relation to the association between the maternal genetically predicted CHD risk and other APOs, pregnancy loss is a heterogeneous condition, and maternal predisposition to cardiovascular disease may only be relevant to specific subsets of losses. Stillbirth, which was suggestively linked to greater genetic liability for CHD in our study, is thought to be induced by placental insufficiency and fetal growth restrictions due to some cardiovascular risk factors during pregnancy, such as hypertension [36–38]. Our findings also revealed an unexpected inverse association between the GRS for CHD and LGA. However, given the small magnitude of the relationship in comparison to the other APOs, and the established role of glucose metabolic disturbances (such as type 1 diabetes, GD or prediabetic states due to excess weight) in increasing the risk of having LGA babies [39–41], it would be advisable to validate the association between the GRS for CHD and LGA in future studies involving other populations. As for GD, it has been shown to be closely related to type 2 diabetes, including with a recent GWAS showing strong genetic correlation between the two [42]. Given the associations of type 2 and GD with cardiovascular disease [43, 44], it is surprising that we did not see an association of the maternal GRS for CHD with GD in our study. Limited cases in our population (~ 2%), the between-study heterogeneity in MoBa and HUNT for this outcome, changes in diagnostic criteria for GD in Norway (which were large and occurred within the time frame of both studies) [28], and underrepresentation in the early years of the MBRN (the precise definition of GD, which differentiates between the diabetic status of the pregnant mother before and during pregnancy, was introduced in the MBRN registration forms in December 1998) [45] may contribute to systematic errors in its diagnosis and hinder our ability to find robust associations. Finally, the lack of association between maternal genetically predicted CHD and sPTB may be due to the fact that not all risk factors for this condition are cardiovascular-related [46].

We conceptualized paternal genetic CHD risk as an imperfect negative control, as an association between the paternal GRS for CHD and APO risk in female partners could not arise from a direct stress test of pregnancy but associations might arise from shared family environment, paternally transmitted genetic variants of the fetus [47] or a maternal immunological response to pregnancy [48]. The mutual adjustment of paternal for maternal GRS should limit the effect of shared family environment, making this an unlikely explanation for the association between the paternal GRS for CHD and risk of sPTB in female partners. There is evidence of both maternal and paternal genetic effects on gestational duration [49]. However, this is not consistent with other of our findings (no strong evidence of a relationship between maternal GRS and sPTB). Given the number of associations that we have explored, and the different association observed in MoBa and HUNT (affected by between-study heterogeneity), it is possible that the association of paternal GRS for CHD with sPTB in female partners is a chance finding, which we would suggest treating with caution unless replicated in other independent studies. Of note, the relationships between the maternal GRS for CHD and HDP, preeclampsia, SGA, and stillbirth were not attenuated after adjusting for the male partners’ GRS for CHD, suggesting that these relationships are specific to women and supporting these APOs likely reflecting pre-existing cardiovascular risk.

Our study has some limitations. Firstly, male partners were considered as imperfect negative controls in our study, mainly due to the shared family environment that could lead to associations between paternal GRS for CHD and APOs in female partners. However, the mutual adjustment of maternal and paternal exposures in negative control analyses aims to control for these shared factors, including those occurring by assortative mating [34]. This imperfection may also be due to the fact that paternal genetic variants related to CHD could affect their reproductive cells’ epigenetics and quality and may impact certain placental characteristics that depend on the fetal genotype, which might subsequently alter the risk of APOs in female partners by alternative mechanisms [15–18]. Secondly, we identified differences between MoBa and HUNT participants with and without genotype data, which could result in selection bias and reduce our external validity. Differences in MoBa might stem from blood sampling during pregnancy or at delivery and it could be less likely if APOs had been detected. This could lead to an understated link between GRS for CHD and APOs due to a possible underrepresentation of APO cases. In HUNT, genotyped participants were older. Longer follow-up time could be associated with a greater probability of an APO diagnosis, possibly inflating the association between the GRS and APOs. However, selection bias had minimal impact on pregnancy outcomes when comparing MoBa with the general Norwegian population [50]. Thirdly, there is risk of potential misclassification for certain APOs in our study. Prior research using MBRN data indicated that the positive predictive value and specificity of preeclampsia diagnosis were satisfactory while the sensitivity was low [51], a scenario that leads to high accuracy for true positives but a certain risk of misclassification due to false negatives. Regarding GD, its comparatively low prevalence, the observed prevalence discrepancies between the MoBa and HUNT studies, alterations in its diagnostic criteria in Norway during the study period, and the particularly low prevalence noted until 1998, may all have hampered the validity of its diagnosis [28, 45]. In both cases, the lack of maternal blood pressure, proteinuria, or glucose level measurements prevents the validation of these diagnoses in our data. Fourthly, we could not calculate sex-specific GRSs for CHD exposures due to the lack of sex-specific results in the largest GWAS on CHD. We assumed no sex differences in the GRS for CHD, but if untrue, our findings might be biased. The bias direction is uncertain without clear gender-based genetic risk information. Fifthly, our findings were not validated in cohorts that are more ethnically heterogeneous and should be interpreted with caution when considering their application to ethnically diverse populations. Lastly, our study population’s characteristics (predominantly adult European women and men) limit the generalizability of our findings to other populations. Despite these limitations, our study has several strengths. To our knowledge, it is the first to investigate the role of genetic liability for CHD in APOs, conducted in a large, well-characterized, and genetically homogeneous population. This complements previous studies that have shown associations of cardiovascular risk factors (e.g., high blood pressure, dyslipidemia, impaired glucose tolerance) with APOs [2–7], by using a GRS that captures environmental (i.e., genetic risk of higher body mass, smoking, education) as well as likely biological risk for CHD. Unlike those previous studies, interpretation of the magnitude of one SD difference in a genetic risk score for CHD is unclear. For example, the relative 8% increased odds for HDP and preeclampsia (OR 1.08, for both) appears small but must be understood in the context that the genetic variants that we used in the GRS explain ~ 15% of the variation in CHD risk. We interpret our results as a qualitative indication of the association between a propensity to CHD influencing the risk of APOs, rather than trying to quantify that relationship.

## Conclusions

Our results indicate that APOs may unmask or exacerbate a woman’s pre-existing cardiovascular risk and suggest that a family history of cardiovascular disease or early onset of cardiovascular outcomes could help identify individuals who might benefit from advice and closer monitoring before and during pregnancy.

### Supplementary Information

**Additional file 1: Table S1. **Genetic variants used in the calculation of the genetic risk score for coronary heart disease. **Table S2. **Description of participants with and without genotype data in MoBa and HUNT. **Table S3. **Sample sizes. **Figure S1.** Associations between one SD higher maternal genetic risk score for coronary heart disease and adverse pregnancy outcomes (A) and between one SD higher paternal genetic risk score and adverse pregnancy outcomes in female partners (B), in MoBa and HUNT participants individually.

## Data Availability

The consent given by the MoBa and HUNT participants does not allow for storage of individual data in repositories or journals. Researchers who want to access MoBa datasets for replication should apply by sending an e-mail to datatilgang@fhi.no. This procedure requires approval from the Regional Committee for Medical and Health Research Ethics in Norway and an agreement with MoBa. Data from the HUNT Study used for research are available upon request to the HUNT Data Access Committee (hunt@medisin.ntnu.no) to research groups who meet the data availability requirements described in http://www.ntnu.edu/hunt/data. Source data of the GWAS on CHD are available in the Online Table VI of the article of Van Der Harst P et al., Circ Res, 2018, available in the Supplemental Materials (https://www.ahajournals.org/doi/suppl/10.1161/CIRCRESAHA.117.312086), as well as in the IEU Open GWAS website (code: ebi-a-GCST005195).

## Abbreviations

APO: Adverse pregnancy outcome
CHD: Coronary heart disease
CI: Confidence interval
GD: Gestational diabetes
GRS: Genetic risk score
GWAS: Genome-wide association study
HDP: Hypertensive disorders of pregnancy
HUNT: Trøndelag Health Study
LGA: Large for gestational age
MBRN: Medical Birth Registry of Norway
MoBa: Mother, Father, and Child Cohort Study
OR: Odds ratio
SD: Standard deviation
SGA: Small for gestational age
sPTB: Spontaneous preterm birth

## References

[1] Sattar N, Greer IA (2002). Pregnancy complications and maternal cardiovascular risk: opportunities for intervention and screening?. BMJ.

[2] Egeland GM, Klungsøyr K, Øyen N, Tell GS, Næss Ø, Skjærven R (2016). Preconception Cardiovascular Risk Factor Differences Between Gestational Hypertension and Preeclampsia: Cohort Norway Study. Hypertension.

[3] Retnakaran R, Shah BR (2020). Divergent Trajectories of Cardiovascular Risk Factors in the Years Before Pregnancy in Women With and Without Gestational Diabetes Mellitus: A Population-Based Study. Diabetes Care.

[4] Foo FL, Mahendru AA, Masini G, Fraser A, Cacciatore S, MacIntyre DA, McEniery CM, Wilkinson IB, Bennett PR, Lees CC (2018). Association Between Prepregnancy Cardiovascular Function and Subsequent Preeclampsia or Fetal Growth Restriction. Hypertension.

[5] Haug EB, Horn J, Markovitz AR, Fraser A, Vatten LJ, Macdonald-Wallis C, Tilling K, Romundstad PR, Rich-Edwards JW, Åsvold BO (2018). Life Course Trajectories of Cardiovascular Risk Factors in Women With and Without Hypertensive Disorders in First Pregnancy: The HUNT Study in Norway. J Am Heart Assoc.

[6] Horn J, Haug EB, Markovitz AR, Fraser A, Vatten LJ, Romundstad PR, Rich-Edwards JW, Åsvold BO (2020). Life Course Trajectories of Maternal Cardiovascular Risk Factors according to Offspring Birthweight: The HUNT Study. Sci Rep.

[7] Markovitz AR, Haug EB, Horn J, Fraser A, Tilling K, Rimm EB, Missmer SA, Williams PL, Romundstad PR, Åsvold BO (2021). Normotensive preterm delivery and maternal cardiovascular risk factor trajectories across the life course: The HUNT Study. Norway Acta Obstet Gynecol Scand.

[8] Wang Q, Würtz P, Auro K, Mäkinen VP, Kangas AJ, Soininen P, Tiainen M, Tynkkynen T, Jokelainen J, Santalahti K (2016). Metabolic profiling of pregnancy: cross-sectional and longitudinal evidence. BMC Med.

[9] Stuart JJ, Tanz LJ, Rimm EB, Spiegelman D, Missmer SA, Mukamal KJ, Rexrode KM, Rich-Edwards JW (2022). Cardiovascular Risk Factors Mediate the Long-Term Maternal Risk Associated With Hypertensive Disorders of Pregnancy. J Am Coll Cardiol.

[10] Tobias DK, Stuart JJ, Li S, Chavarro J, Rimm EB, Rich-Edwards J, Hu FB, Manson JE, Zhang C (2017). Association of History of Gestational Diabetes With Long-term Cardiovascular Disease Risk in a Large Prospective Cohort of US Women. JAMA Intern Med.

[11] Romundstad PR, Magnussen EB, Smith GD, Vatten LJ. Hypertension in pregnancy and later cardiovascular risk: common antecedents? 2010(1524–4539 (Electronic)). 10.1161/CIRCULATIONAHA.110.943407 20660802

[12] ACOG Practice Bulletin No (2019). 202: Gestational Hypertension and Preeclampsia. Obstet Gynecol.

[13] Honigberg MC, Truong B, Khan RR, Xiao B, Bhatta L, Vy HMT, Guerrero RF, Schuermans A, Selvaraj MS, Patel AP, et al. Polygenic prediction of preeclampsia and gestational hypertension. Nat Med. 2023;29(6):1540–9. 10.1038/s41591-023-02374-9 PMC10330886 37248299

[14] Borges MC, Clayton G, Freathy RM, Felix JF, Fernández-Sanlés A, Gonçalves Soares A, Kilpi F, Yang Q, McEachan RRC, Richmond RC, et al. Integrating multiple lines of evidence to assess the effects of maternal BMI on pregnancy and perinatal outcomes in up to 497,932 women. medRxiv. 2022:2022.2007.2022.22277930. 10.1186/s12916-023-03167-0 PMC10823651 38281920

[15] Galaviz-Hernandez C, Sosa-Macias M, Teran E, Garcia-Ortiz JE, Lazalde-Ramos BP (2018). Paternal Determinants in Preeclampsia. Front Physiol.

[16] Sharp GC, Lawlor DA (2019). Paternal impact on the life course development of obesity and type 2 diabetes in the offspring. Diabetologia.

[17] Beaumont RN, Flatley C, Vaudel M, Wu X, Chen J, Moen G-H, Skotte L, Helgeland Ø, Sole-Navais P, Banasik K, et al. Genome-wide association study of placental weight in 179,025 children and parents reveals distinct and shared genetic influences between placental and fetal growth. medRxiv. 2022:2022.2011.2025.22282723.

[18] Tyrmi JS, Kaartokallio T, Lokki AI, Jääskeläinen T, Kortelainen E, Ruotsalainen S, Karjalainen J, Ripatti S, Kivioja A, Laisk T, et al. Genetic Risk Factors Associated With Preeclampsia and Hypertensive Disorders of Pregnancy. JAMA Cardiol. 2023;8(7):674–83. 10.1001/jamacardio.2023.1312 PMC10248811 37285119

[19] van der Harst P, Verweij N (2018). Identification of 64 Novel Genetic Loci Provides an Expanded View on the Genetic Architecture of Coronary Artery Disease. Circ Res.

[20] Magnus P, Birke C, Vejrup K, Haugan A, Alsaker E, Daltveit AK, Handal M, Haugen M, Høiseth G, Knudsen GP (2016). Cohort Profile Update: The Norwegian Mother and Child Cohort Study (MoBa). Int J Epidemiol.

[21] Åsvold BO, Langhammer A, Rehn TA, Kjelvik G, Grøntvedt TV, Sørgjerd EP, Fenstad JS, Heggland J, Holmen O, Stuifbergen MC et al: Cohort Profile Update: The HUNT Study, Norway. Int J Epidemiol. 2022;52(1):e80–91. 10.1093/ije/dyac095 PMC9908054 35578897

[22] Brumpton BM, Graham S, Surakka I, Skogholt AH, Løset M, Fritsche LG, Wolford B, Zhou W, Nielsen JB, Holmen OL, et al. The HUNT Study: a population-based cohort for genetic research. Cell Genom. 2022;2(10):100193. 10.1016/j.xgen.2022.100193 PMC9903730 36777998

[23] Irgens LM (2000). The Medical Birth Registry of Norway. Epidemiological research and surveillance throughout 30 years. Acta Obstet Gynecol Scand..

[24] Corfield EC, Frei O, Shadrin AA, Rahman Z, Lin A, Athanasiu L, Akdeniz BC, Hannigan L, Wootton RE, Austerberry C, et al. The Norwegian Mother, Father, and Child cohort study (MoBa) genotyping data resource: MoBaPsychGen pipeline v.1. bioRxiv. 2022.

[25] SNPs for coronary artery disease (Dataset: ebi-a-GCST005195). https://gwas.mrcieu.ac.uk/datasets/ebi-a-GCST005195/. Accessed 18 Aug 2023.

[26] Choi SW, Mak TS, O’Reilly PF. Tutorial: a guide to performing polygenic risk score analyses. Nat Protoc. 2020;15(9):2759–72. 10.1038/s41596-020-0353-1 PMC7612115 32709988

[27] Scott G, Gillon TE, Pels A, von Dadelszen P, Magee LA (2022). Guidelines-similarities and dissimilarities: a systematic review of international clinical practice guidelines for pregnancy hypertension. Am J Obstet Gynecol.

[28] Norwegian Guidelines for gestational diabetes (Svangerskapsdiabetes). https://www.legeforeningen.no/foreningsledd/fagmed/norsk-gynekologisk-forening/veiledere/veileder-i-fodselshjelp/svangerskapsdiabetes/. Accessed 18 Aug 2023.

[29] Reforma LG, Febres-Cordero D, Trochtenberg A, Modest AM, Collier AY, Spiel MH. Incidence of small-for-gestational-age infant birthweight following early intertwin fetal growth discordance in dichorionic and monochorionic twin pregnancies. Am J Obstet Gynecol. 2022;226(5):726.e1–726.e9. 10.1016/j.ajog.2021.11.1358 PMC9064885 34838799

[30] Reddy UM, Deshmukh U, Dude A, Harper L, Osmundson SS (2021). Society for Maternal-Fetal Medicine Consult Series #58: Use of antenatal corticosteroids for individuals at risk for late preterm delivery: Replaces SMFM Statement #4, Implementation of the use of antenatal corticosteroids in the late preterm birth period in women at risk for preterm delivery, August 2016. Am J Obstet Gynecol.

[31] Skåra KH, Åsvold BO, Hernáez Á, Fraser A, Rich-Edwards JW, Farland LV, Næss Ø, Lawlor DA, Brumpton B, Magnus MC. Risk of cardiovascular disease in women and men with subfertility: the Trøndelag Health Study. Fertil Steril. 2022;118(3):537–47. 10.1016/j.fertnstert.2022.05.038 35840354

[32] Hernáez Á, Wootton RE, Page CM, Skåra KH, Fraser A, Rogne T, Magnus P, Njølstad PR, Andreassen OA, Burgess S, et al. Smoking and infertility: multivariable regression and Mendelian randomization analyses in the Norwegian Mother, Father and Child Cohort Study. Fertil Steril. 2022;118(1):180–90. 10.1016/j.fertnstert.2022.04.001 PMC7612999 35562204

[33] Burgess S, Davey Smith G, Davies NM, Dudbridge F, Gill D, Glymour MM, Hartwig FP, Holmes MV, Minelli C, Relton CL (2019). Guidelines for performing Mendelian randomization investigations. Wellcome Open Res.

[34] Madley-Dowd P, Rai D, Zammit S, Heron J (2020). Simulations and directed acyclic graphs explained why assortative mating biases the prenatal negative control design. J Clin Epidemiol.

[35] Ardissino M, Slob EAW, Millar O, Reddy RK, Lazzari L, Patel KHK, Ryan D, Johnson MR, Gill D, Ng FS (2022). Maternal Hypertension Increases Risk of Preeclampsia and Low Fetal Birthweight: Genetic Evidence From a Mendelian Randomization Study. Hypertension.

[36] Brosens I, Pijnenborg R, Vercruysse L, Romero R (2011). The, “Great Obstetrical Syndromes” are associated with disorders of deep placentation. Am J Obstet Gynecol..

[37] Page JM, Silver RM (2017). Interventions to prevent stillbirth. Semin Fetal Neonatal Med.

[38] Nobles CJ, Mendola P, Mumford SL, Naimi AI, Yeung EH, Kim K, Park H, Wilcox B, Silver RM, Perkins NJ (2018). Preconception Blood Pressure Levels and Reproductive Outcomes in a Prospective Cohort of Women Attempting Pregnancy. Hypertension.

[39] Black MH, Sacks DA, Xiang AH, Lawrence JM (2013). The relative contribution of prepregnancy overweight and obesity, gestational weight gain, and IADPSG-defined gestational diabetes mellitus to fetal overgrowth. Diabetes Care.

[40] Landon MB, Spong CY, Thom E, Carpenter MW, Ramin SM, Casey B, Wapner RJ, Varner MW, Rouse DJ, Thorp JM (2009). A multicenter, randomized trial of treatment for mild gestational diabetes. N Engl J Med.

[41] Persson M, Norman M, Hanson U (2009). Obstetric and perinatal outcomes in type 1 diabetic pregnancies: A large, population-based study. Diabetes Care.

[42] Pervjakova N, Moen GH, Borges MC, Ferreira T, Cook JP, Allard C, Beaumont RN, Canouil M, Hatem G, Heiskala A, et al. Multi-ancestry genome-wide association study of gestational diabetes mellitus highlights genetic links with type 2 diabetes. Hum Mol Genet. 2022;31(19):3377–91. 10.1093/hmg/ddac050 PMC9523562 35220425

[43] Parikh NI, Gonzalez JM, Anderson CAM, Judd SE, Rexrode KM, Hlatky MA, Gunderson EP, Stuart JJ, Vaidya D (2021). Adverse Pregnancy Outcomes and Cardiovascular Disease Risk: Unique Opportunities for Cardiovascular Disease Prevention in Women: A Scientific Statement From the American Heart Association. Circulation.

[44] Sarwar N, Gao P, Seshasai SR, Gobin R, Kaptoge S, Di Angelantonio E, Ingelsson E, Lawlor DA, Selvin E, Stampfer M (2010). Diabetes mellitus, fasting blood glucose concentration, and risk of vascular disease: a collaborative meta-analysis of 102 prospective studies. Lancet.

[45] Stene LC, Eidem I, Vangen S, Joner G, Irgens LM, Moe N. The validity of the diabetes mellitus diagnosis in the Medical Birth Registry of Norway. Norsk Epidemiologi. 2007;17(2):165–74.

[46] Kemp MW (2014). Preterm birth, intrauterine infection, and fetal inflammation. Front Immunol.

[47] Shah PS (2010). Paternal factors and low birthweight, preterm, and small for gestational age births: a systematic review. Am J Obstet Gynecol.

[48] Gomez-Lopez N, Galaz J, Miller D, Farias-Jofre M, Liu Z, Arenas-Hernandez M, Garcia-Flores V, Shaffer Z, Greenberg JM, Theis KR (2022). The immunobiology of preterm labor and birth: intra-amniotic inflammation or breakdown of maternal-fetal homeostasis. Reproduction.

[49] Lie RT, Wilcox AJ, Skjaerven R (2006). Maternal and paternal influences on length of pregnancy. Obstet Gynecol.

[50] Nilsen RM, Vollset SE, Gjessing HK, Skjaerven R, Melve KK, Schreuder P, Alsaker ER, Haug K, Daltveit AK, Magnus P (2009). Self-selection and bias in a large prospective pregnancy cohort in Norway. Paediatr Perinat Epidemiol.

[51] Klungsøyr K, Harmon QE, Skard LB, Simonsen I, Austvoll ET, Alsaker ER, Starling A, Trogstad L, Magnus P, Engel SM (2014). Validity of pre-eclampsia registration in the medical birth registry of Norway for women participating in the Norwegian mother and child cohort study, 1999–2010. Paediatr Perinat Epidemiol.

