# Supplementary material for: Parental genetically predicted liability for coronary heart disease and risk of adverse pregnancy outcomes: a cohort study
Source: BMC Med. 2024 Jan 25;22:35. doi: 10.1186/s12916-023-03223-9 (PMC10809500; doi:10.1186/s12916-023-03223-9)
Supplement: Supplementary file 1 — Additional file 1: Table S1. Genetic variants used in the calculation of the genetic risk score for coronary heart disease. Table S2. Description of participants with and without genotype data in MoBa and HUNT. Table S3. Sample sizes. Figure S1. Associations between one SD higher maternal genetic risk score for coronary heart disease and adverse pregnancy outcomes (A) and between one SD higher paternal genetic risk score and adverse pregnancy outcomes in female partners (B), in MoBa and HUNT participants individually. [file 12916_2023_3223_MOESM1_ESM.docx]

**ADDITIONAL FILE 1**

**SUPPLEMENTARY TABLES**

**Table S1.** Genetic variants used in the calculation of the genetic risk score for coronary heart disease.

| RSID | Chromosome | Position | Reference  allele | Effect  allele | Effect allele  frequency | Beta | Standard  error | *p*-value | Available  in MoBa | Available  in HUNT |
| --- | --- | --- | --- | --- | --- | --- | --- | --- | --- | --- |
| rs36096196 | 1 | 2252205 | C | T | 0.1483 | 0.0469 | 0.0082 | 1.33E-08 | Yes | Yes |
| rs2493298 | 1 | 3325912 | C | A | 0.1355 | 0.0514 | 0.0084 | 9.97E-10 | Yes | Yes |
| rs4072980 | 1 | 38456106 | G | A | 0.4303 | -0.0326 | 0.0052 | 4.13E-10 | Yes | Yes |
| rs11591147 | 1 | 55505647 | G | T | 0.0164 | -0.2406 | 0.0247 | 1.86E-22 | No | Yes |
| rs17114046 | 1 | 56966350 | G | A | 0.9062 | 0.096 | 0.0088 | 8.04E-28 | Yes | Yes |
| rs602633 | 1 | 109821511 | G | T | 0.2343 | -0.0999 | 0.0062 | 3.63E-58 | Yes | Yes |
| rs11806316 | 1 | 115753482 | G | A | 0.3668 | -0.0337 | 0.0054 | 4.16E-10 | Yes | Yes |
| rs11204892 | 1 | 151768048 | G | A | 0.1892 | -0.0487 | 0.0073 | 2.85E-11 | Yes | Yes |
| rs4845625 | 1 | 154422067 | C | T | 0.4338 | 0.0395 | 0.0051 | 6.69E-15 | Yes | Yes |
| rs1892094 | 1 | 169094459 | C | T | 0.4956 | -0.0362 | 0.0052 | 3.14E-12 | Yes | Yes |
| rs6700559 | 1 | 200646073 | C | T | 0.4715 | -0.0284 | 0.005 | 1.77E-08 | Yes | Yes |
| rs2820315 | 1 | 201872264 | C | T | 0.3012 | 0.0359 | 0.0056 | 1.10E-10 | Yes | Yes |
| rs60154123 | 1 | 210468999 | C | T | 0.1501 | 0.0444 | 0.008 | 2.58E-08 | Yes | Yes |
| rs35158675 | 1 | 222829550 | G | A | 0.6821 | 0.0699 | 0.0064 | 5.91E-28 | Yes | Yes |
| rs16986953 | 2 | 19942473 | G | A | 0.1095 | 0.0812 | 0.0098 | 1.06E-16 | Yes | Yes |
| rs515135 | 2 | 21286057 | C | T | 0.1837 | -0.0555 | 0.0066 | 5.74E-17 | Yes | Yes |
| rs6544713 | 2 | 44073881 | C | T | 0.3038 | 0.0492 | 0.0056 | 1.84E-18 | Yes | Yes |
| rs582384 | 2 | 45896437 | C | A | 0.5251 | 0.0332 | 0.0058 | 7.64E-09 | Yes | Yes |
| rs6743030 | 2 | 85763520 | C | T | 0.465 | 0.0565 | 0.0057 | 1.81E-23 | Yes | Yes |
| rs2252641 | 2 | 145801461 | C | T | 0.5299 | -0.0368 | 0.0051 | 5.41E-13 | Yes | Yes |
| rs12999907 | 2 | 164957251 | G | A | 0.8189 | 0.0482 | 0.0074 | 6.31E-11 | Yes | Yes |
| rs6728861 | 2 | 203873743 | G | A | 0.1174 | 0.1054 | 0.0089 | 1.29E-32 | Yes | Yes |
| rs1250229 | 2 | 216304384 | C | T | 0.2722 | 0.0435 | 0.0059 | 1.58E-13 | Yes | Yes |
| rs2571445 | 2 | 218683154 | G | A | 0.3895 | 0.037 | 0.0052 | 1.56E-12 | Yes | Yes |
| rs11677932 | 2 | 238223955 | G | A | 0.3157 | -0.0339 | 0.0061 | 2.64E-08 | Yes | Yes |
| rs34991912 | 3 | 14926351 | C | T | 0.4344 | 0.0379 | 0.0057 | 3.21E-11 | Yes | Yes |
| rs7633770 | 3 | 46688562 | G | A | 0.4061 | 0.0295 | 0.0052 | 1.10E-08 | Yes | Yes |
| rs7617773 | 3 | 48193515 | C | T | 0.6693 | 0.0355 | 0.0054 | 3.48E-11 | Yes | Yes |
| rs4678145 | 3 | 124450081 | G | C | 0.1284 | 0.0639 | 0.0083 | 1.45E-14 | Yes | Yes |
| rs10512861 | 3 | 132257961 | G | T | 0.1433 | -0.0431 | 0.0073 | 3.00E-09 | Yes | Yes |
| rs667920 | 3 | 136069472 | G | T | 0.7751 | 0.0489 | 0.0063 | 6.08E-15 | Yes | Yes |
| rs185244 | 3 | 138092889 | C | T | 0.1625 | 0.0644 | 0.0076 | 2.40E-17 | Yes | Yes |
| rs789294 | 3 | 154057741 | G | C | 0.1649 | -0.0642 | 0.0081 | 3.36E-15 | Yes | Yes |
| rs4266144 | 3 | 156852592 | G | C | 0.6774 | -0.0346 | 0.0061 | 1.40E-08 | Yes | Yes |
| rs12897 | 3 | 172115902 | G | A | 0.5914 | -0.0358 | 0.0059 | 1.21E-09 | Yes | Yes |
| rs17081933 | 4 | 57822869 | T | A | 0.1979 | 0.0399 | 0.007 | 1.17E-08 | Yes | Yes |
| rs12500824 | 4 | 77416627 | G | A | 0.3565 | 0.0336 | 0.0053 | 1.64E-10 | Yes | Yes |
| rs10857147 | 4 | 81181072 | T | A | 0.7208 | -0.0491 | 0.0064 | 1.37E-14 | Yes | Yes |
| rs11099493 | 4 | 82587050 | G | A | 0.6924 | 0.039 | 0.0062 | 2.49E-10 | Yes | Yes |
| rs3775058 | 4 | 96117371 | T | A | 0.2341 | 0.0386 | 0.0067 | 7.63E-09 | Yes | Yes |
| rs7678555 | 4 | 120909501 | C | A | 0.7166 | -0.0486 | 0.0063 | 1.23E-14 | Yes | Yes |
| rs6841581 | 4 | 148401190 | G | A | 0.1559 | 0.0751 | 0.0074 | 5.18E-24 | Yes | Yes |
| rs7692387 | 4 | 156635309 | G | A | 0.1892 | -0.0643 | 0.0065 | 3.34E-23 | Yes | Yes |
| rs7696431 | 4 | 169687725 | G | T | 0.5098 | 0.0311 | 0.0056 | 3.09E-08 | Yes | Yes |
| rs112941079 | 5 | 9546098 | G | A | 0.8676 | 0.0615 | 0.0085 | 3.75E-13 | Yes | Yes |
| rs3936511 | 5 | 55860781 | G | A | 0.823 | -0.0366 | 0.0067 | 3.74E-08 | Yes | Yes |
| rs273909 | 5 | 131667353 | G | A | 0.8754 | -0.0488 | 0.0078 | 4.74E-10 | No | Yes |
| rs246600 | 5 | 142516897 | C | T | 0.4633 | 0.043 | 0.0051 | 6.53E-17 | Yes | Yes |
| rs9349379 | 6 | 12903957 | G | A | 0.5907 | -0.1072 | 0.0058 | 2.72E-76 | Yes | Yes |
| rs6909752 | 6 | 22612629 | G | A | 0.3545 | 0.0442 | 0.0059 | 5.71E-14 | Yes | Yes |
| rs644045 | 6 | 31883957 | G | A | 0.3528 | -0.0413 | 0.0057 | 2.80E-13 | Yes | No |
| rs2814944 | 6 | 34552797 | G | A | 0.1554 | 0.0479 | 0.0072 | 2.15E-11 | Yes | Yes |
| rs1334894 | 6 | 35615130 | C | T | 0.0919 | -0.0482 | 0.0088 | 4.84E-08 | Yes | Yes |
| rs1321309 | 6 | 36638636 | G | A | 0.4907 | 0.028 | 0.0051 | 3.48E-08 | Yes | Yes |
| rs10947789 | 6 | 39174922 | C | T | 0.7671 | 0.0413 | 0.006 | 7.63E-12 | Yes | Yes |
| rs6905288 | 6 | 43758873 | G | A | 0.5807 | 0.0386 | 0.0056 | 4.00E-12 | Yes | Yes |
| rs71566846 | 6 | 57148171 | C | T | 0.0662 | 0.0829 | 0.0114 | 3.24E-13 | Yes | Yes |
| rs4613862 | 6 | 82612271 | C | A | 0.5314 | 0.0319 | 0.0052 | 6.58E-10 | Yes | Yes |
| rs1591805 | 6 | 126717064 | G | A | 0.4927 | 0.037 | 0.0058 | 1.17E-10 | Yes | Yes |
| rs2327429 | 6 | 134209837 | C | T | 0.695 | 0.0655 | 0.0056 | 3.56E-31 | Yes | Yes |
| rs17080091 | 6 | 150997401 | C | T | 0.0839 | -0.0537 | 0.0092 | 6.09E-09 | Yes | Yes |
| rs55730499 | 6 | 161005610 | C | T | 0.0693 | 0.3122 | 0.0118 | 9.78E-154 | Yes | Yes |
| rs10267593 | 7 | 1937261 | G | A | 0.2012 | -0.036 | 0.0064 | 1.88E-08 | Yes | Yes |
| rs7797644 | 7 | 6486067 | C | T | 0.2321 | -0.0386 | 0.0069 | 2.10E-08 | Yes | Yes |
| rs11509880 | 7 | 12261911 | G | A | 0.3612 | 0.0327 | 0.0059 | 3.27E-08 | Yes | Yes |
| rs2107595 | 7 | 19049388 | G | A | 0.1767 | 0.0752 | 0.0073 | 1.25E-24 | Yes | Yes |
| rs2107732 | 7 | 45077978 | G | A | 0.0874 | -0.0567 | 0.0103 | 3.64E-08 | No | Yes |
| rs2189839 | 7 | 107230026 | G | A | 0.2899 | 0.0345 | 0.0063 | 4.53E-08 | Yes | Yes |
| rs975722 | 7 | 117332914 | G | A | 0.595 | -0.0283 | 0.0052 | 4.17E-08 | Yes | Yes |
| rs11556924 | 7 | 129663496 | C | T | 0.3624 | -0.0548 | 0.0055 | 1.37E-23 | Yes | Yes |
| rs10237377 | 7 | 139757136 | G | T | 0.3608 | -0.0338 | 0.0058 | 6.53E-09 | Yes | Yes |
| rs3918226 | 7 | 150690176 | C | T | 0.0732 | 0.1071 | 0.0115 | 1.35E-20 | Yes | Yes |
| rs6997340 | 8 | 18286997 | C | T | 0.3025 | 0.0331 | 0.0056 | 4.60E-09 | Yes | Yes |
| rs17091891 | 8 | 19843171 | C | T | 0.8709 | 0.0585 | 0.0078 | 5.15E-14 | Yes | Yes |
| rs6984210 | 8 | 22033615 | G | C | 0.939 | -0.0784 | 0.0115 | 1.04E-11 | No | Yes |
| rs10093110 | 8 | 106565414 | G | A | 0.4167 | -0.0319 | 0.0057 | 1.88E-08 | Yes | Yes |
| rs6982502 | 8 | 126479362 | C | T | 0.5298 | -0.0498 | 0.0051 | 7.67E-23 | Yes | Yes |
| rs4977574 | 9 | 22098574 | G | A | 0.519 | -0.1788 | 0.0056 | 8.82E-223 | Yes | Yes |
| rs944172 | 9 | 110517794 | C | T | 0.7197 | -0.0395 | 0.0057 | 3.59E-12 | Yes | Yes |
| rs885150 | 9 | 124420173 | C | T | 0.7304 | -0.0355 | 0.0058 | 7.86E-10 | Yes | Yes |
| rs2519093 | 9 | 136141870 | C | T | 0.1841 | 0.0554 | 0.0072 | 2.03E-14 | Yes | Yes |
| rs61848342 | 10 | 12303813 | C | T | 0.6355 | -0.0363 | 0.0059 | 6.38E-10 | Yes | Yes |
| rs9337951 | 10 | 30317073 | G | A | 0.3178 | 0.0543 | 0.0064 | 1.73E-17 | No | Yes |
| rs1870634 | 10 | 44480811 | G | T | 0.3511 | -0.06 | 0.0059 | 3.50E-24 | Yes | Yes |
| rs17680741 | 10 | 82251514 | C | T | 0.7152 | 0.042 | 0.0062 | 1.72E-11 | Yes | Yes |
| rs1412444 | 10 | 91002927 | C | T | 0.3527 | 0.0559 | 0.0059 | 2.43E-21 | Yes | Yes |
| rs3740390 | 10 | 104638480 | C | T | 0.1066 | -0.0662 | 0.0084 | 4.67E-15 | Yes | Yes |
| rs4918072 | 10 | 105693644 | G | A | 0.2713 | 0.0386 | 0.0063 | 9.63E-10 | Yes | Yes |
| rs4752700 | 10 | 124237612 | G | A | 0.5508 | -0.0332 | 0.0051 | 8.02E-11 | Yes | Yes |
| rs11601507 | 11 | 5701074 | C | A | 0.0739 | 0.0782 | 0.0108 | 5.61E-13 | Yes | Yes |
| rs472109 | 11 | 9770318 | G | C | 0.5698 | 0.0391 | 0.0057 | 6.26E-12 | Yes | Yes |
| rs7926712 | 11 | 13303085 | G | A | 0.3101 | -0.0351 | 0.0061 | 9.41E-09 | Yes | Yes |
| rs7116641 | 11 | 43696917 | G | T | 0.6877 | -0.0314 | 0.0055 | 1.03E-08 | Yes | Yes |
| rs12801636 | 11 | 65391317 | G | A | 0.2364 | -0.0403 | 0.006 | 2.29E-11 | Yes | Yes |
| rs606452 | 11 | 75276178 | C | A | 0.1707 | -0.0466 | 0.0071 | 4.76E-11 | Yes | Yes |
| rs4754698 | 11 | 100631908 | G | C | 0.5399 | -0.0368 | 0.0056 | 6.79E-11 | Yes | Yes |
| rs974819 | 11 | 103660567 | C | T | 0.3082 | 0.0614 | 0.0055 | 1.12E-28 | Yes | Yes |
| rs651821 | 11 | 116662579 | C | T | 0.901 | -0.0692 | 0.0096 | 7.03E-13 | Yes | Yes |
| rs11838267 | 12 | 7175872 | C | T | 0.8679 | 0.0514 | 0.0083 | 6.15E-10 | No | Yes |
| rs10841443 | 12 | 20220033 | G | C | 0.3409 | -0.046 | 0.0061 | 2.86E-14 | Yes | Yes |
| rs11170820 | 12 | 54513915 | G | C | 0.9292 | -0.0832 | 0.0117 | 9.28E-13 | Yes | Yes |
| rs11613352 | 12 | 57792580 | C | T | 0.235 | -0.036 | 0.0062 | 4.92E-09 | Yes | Yes |
| rs2681492 | 12 | 90013089 | C | T | 0.8141 | -0.0558 | 0.0072 | 8.64E-15 | Yes | Yes |
| rs11107903 | 12 | 95507971 | G | A | 0.0768 | -0.0747 | 0.0105 | 1.06E-12 | Yes | Yes |
| rs7137828 | 12 | 111932800 | C | T | 0.5535 | -0.0643 | 0.006 | 3.72E-27 | Yes | Yes |
| rs1169288 | 12 | 121416650 | C | A | 0.6725 | -0.049 | 0.0056 | 1.26E-18 | Yes | Yes |
| rs11057830 | 12 | 125307053 | G | A | 0.1465 | 0.0655 | 0.0075 | 1.91E-18 | Yes | Yes |
| rs9319428 | 13 | 28973621 | G | A | 0.3121 | 0.036 | 0.0055 | 5.36E-11 | Yes | Yes |
| rs7998440 | 13 | 33125206 | G | A | 0.3549 | -0.0383 | 0.0059 | 1.19E-10 | Yes | Yes |
| rs9515203 | 13 | 111049623 | C | T | 0.7392 | 0.0596 | 0.006 | 3.89E-23 | No | Yes |
| rs1317507 | 13 | 113631780 | C | A | 0.2608 | 0.0398 | 0.0058 | 8.21E-12 | Yes | Yes |
| rs2145598 | 14 | 58794001 | G | A | 0.5758 | -0.0283 | 0.0052 | 4.26E-08 | Yes | Yes |
| rs7145159 | 14 | 75583268 | C | T | 0.5234 | -0.0319 | 0.0051 | 3.60E-10 | Yes | Yes |
| rs112635299 | 14 | 94838142 | G | T | 0.0193 | -0.1363 | 0.0222 | 8.44E-10 | Yes | Yes |
| rs8003602 | 14 | 100148961 | C | T | 0.2681 | -0.0539 | 0.0066 | 2.89E-16 | Yes | Yes |
| rs6494488 | 15 | 65024204 | G | A | 0.8117 | 0.0382 | 0.007 | 3.90E-08 | Yes | Yes |
| rs72743461 | 15 | 67441750 | C | A | 0.2211 | -0.0576 | 0.0069 | 5.68E-17 | Yes | Yes |
| rs7173743 | 15 | 79141784 | C | T | 0.5542 | 0.0636 | 0.0051 | 5.48E-36 | Yes | Yes |
| rs1807214 | 15 | 89565257 | C | A | 0.8891 | 0.0639 | 0.0097 | 5.21E-11 | Yes | Yes |
| rs17514846 | 15 | 91416550 | C | A | 0.4605 | 0.0559 | 0.0052 | 9.86E-27 | Yes | Yes |
| rs17581137 | 15 | 96146414 | C | A | 0.754 | 0.0373 | 0.0065 | 1.21E-08 | Yes | Yes |
| rs12149545 | 16 | 56993161 | G | A | 0.3035 | -0.0374 | 0.0062 | 1.19E-09 | Yes | Yes |
| rs1050362 | 16 | 72130815 | C | A | 0.3755 | 0.0352 | 0.0053 | 2.92E-11 | Yes | Yes |
| rs8046696 | 16 | 75442143 | G | T | 0.4311 | -0.0479 | 0.0058 | 1.91E-16 | Yes | Yes |
| rs7199941 | 16 | 81906423 | G | A | 0.3982 | 0.0367 | 0.0051 | 9.36E-13 | Yes | Yes |
| rs7500448 | 16 | 83045790 | G | A | 0.7594 | 0.0557 | 0.0068 | 1.61E-16 | Yes | Yes |
| rs170041 | 17 | 2170216 | C | T | 0.2943 | -0.0469 | 0.0056 | 4.11E-17 | Yes | Yes |
| rs12936587 | 17 | 17543722 | G | A | 0.4379 | -0.032 | 0.0052 | 9.51E-10 | Yes | Yes |
| rs11080107 | 17 | 27938424 | C | T | 0.5146 | -0.0358 | 0.0057 | 2.90E-10 | Yes | Yes |
| rs76954792 | 17 | 30033514 | C | T | 0.2197 | 0.0393 | 0.0069 | 1.19E-08 | Yes | Yes |
| rs2074164 | 17 | 40270238 | G | C | 0.1956 | 0.0423 | 0.0073 | 7.36E-09 | Yes | Yes |
| rs17608766 | 17 | 45013271 | C | T | 0.8632 | -0.0469 | 0.0076 | 8.20E-10 | Yes | No |
| rs62076439 | 17 | 47404628 | G | T | 0.3409 | 0.048 | 0.006 | 1.63E-15 | Yes | Yes |
| rs1476098 | 17 | 59237013 | C | A | 0.2122 | 0.0443 | 0.0072 | 8.51E-10 | Yes | Yes |
| rs9892152 | 17 | 62401965 | C | T | 0.4717 | -0.033 | 0.005 | 6.28E-11 | Yes | Yes |
| rs9964304 | 18 | 47229717 | C | A | 0.716 | -0.0382 | 0.0063 | 1.14E-09 | Yes | Yes |
| rs663640 | 18 | 57846077 | C | T | 0.222 | 0.0383 | 0.0068 | 2.02E-08 | Yes | Yes |
| rs116843064 | 19 | 8429323 | G | A | 0.0202 | -0.1402 | 0.0224 | 3.57E-10 | Yes | Yes |
| rs55791371 | 19 | 11188153 | C | A | 0.8868 | 0.1157 | 0.0092 | 1.93E-36 | Yes | Yes |
| rs7251815 | 19 | 17844942 | G | T | 0.2162 | 0.0511 | 0.0069 | 1.44E-13 | Yes | Yes |
| rs4803455 | 19 | 41851509 | C | A | 0.4954 | -0.0484 | 0.0057 | 2.36E-17 | Yes | Yes |
| rs7412 | 19 | 45412079 | C | T | 0.079 | -0.1368 | 0.011 | 2.14E-35 | Yes | Yes |
| rs867186 | 20 | 33764554 | G | A | 0.8971 | 0.0573 | 0.0084 | 6.84E-12 | Yes | Yes |
| rs6102343 | 20 | 39924279 | G | A | 0.2467 | 0.0372 | 0.0065 | 1.12E-08 | Yes | Yes |
| rs3827066 | 20 | 44586023 | C | T | 0.1437 | 0.0424 | 0.0072 | 4.40E-09 | Yes | Yes |
| rs260020 | 20 | 57714025 | C | T | 0.1309 | 0.0518 | 0.0084 | 7.96E-10 | Yes | Yes |
| rs2832227 | 21 | 30533076 | G | A | 0.8258 | -0.0393 | 0.0067 | 4.16E-09 | Yes | Yes |
| rs28451064 | 21 | 35593827 | G | A | 0.1239 | 0.1083 | 0.009 | 2.58E-33 | Yes | Yes |

**Table S2.** Description of participants with and without genotype data in MoBa and HUNT.

|  | MoBa | | | | HUNT | | | |
| --- | --- | --- | --- | --- | --- | --- | --- | --- |
|  | Women | | Men | | Women | | Men | |
|  | Included | Non-included | Included | Non-included | Included | Non-included | Included | Non-included |
| Birth year,  median (1^st^-3^rd^ quartile) | 1975  (1972-1979) | 1975  (1972-1979) | 1973  (1970-1977) | 1973  (1969-1976) | 1964  (1958-1971) | 1976  (1966-1985) | 1962  (1956-1969) | 1974  (1964-1983) |
| Number of pregnancies, median (1^st^-3^rd^ quartile) | 2  (2-3) | 2  (2-3) | 2  (2-3) | 2  (2-3) | 2  (2-3) | 2  (2-3) | 2  (2-3) | 2  (2-3) |
| Participants in pregnancies with more than one different partner, *n* (%) | 9,147  (13.3%) | 2,099  (13.1%) | 5,819  (12.3%) | 2,945  (13.9%) | 745  (4.74%) | 203  (3.53%) | 178  (2.08%) | 51  (1.70%) |
| Age in first pregnancy,  median (1^st^-3^rd^ quartile) | 27  (24-30) | 27  (24-30) | 29  (26-33) | 29  (26-33) | 24  (20-27) | 24  (21-28) | 26  (23-29) | 27  (24-30) |
| Education years, mean ± SD | 17.0 ± 3.36 | 16.7 ± 3.55 | 16.3 ± 3.56 | 16.0 ± 3.66 | 14.3 ± 4.13 | 15.0 ± 4.14 | 13.5 ± 4.08 | 14.1 ± 4.27 |
| Body mass index (kg/m^2^),  median (1^st^-3^rd^ quartile) | 23.1  (21.1-25.9) | 23.0  (20.9-25.7) | 25.4  (23.6-27.7) | 25.4  (23.6-27.7) | 25.2  (22.7-28.6) | 25.3  (22.6-29.0) | 26.6  (24.5-29.1) | 26.7  (24.5-29.4) |
| Ever smokers, *n* (%) | 35,632  (52.3%) | 8,071  (51.1%) | 24,294  (51.2%) | 11,461  (54.1%) | 8,865  (57.0%) | 3,080  (54.0%) | 4,233  (50.1%) | 1,533  (51.4%) |
| Total number of deliveries, mean ± SD | 2.54 ± 0.85 | 2.55 ± 0.89 | 2.51 ± 0.81 | 2.54 ± 0.86 | 1.48 ± 0.88 | 1.28 ± 0.94 | 1.50 ± 0.81 | 1.31 ± 0.85 |
| Miscarriage, *n* (%) | 20,866  (30.4%) | 4,835  (30.7%) | 14,451  (30.6%) | 6,484  (31.0%) | 1,950  (30.0%)^a^ | 1,184  (29.4%)^a^ | 1,145  (29.6%)^a^ | 662  (29.3%)^a^ |
| Stillbirth, *n* (%) | 872  (1.27%) | 238  (1.51%) | 581  (1.23%) | 313  (1.49%) | 198  (1.30%) | 45  (0.84%) | 84  (0.99%) | 19  (0.64%) |
| Any hypertensive disorders  of pregnancy, *n* (%) | 7,288  (10.6%) | 1,730  (11.0%) | 5,186  (11.0%) | 2,297  (11.0%) | 1,500  (9.87%) | 483  (9.02%) | 870  (10.2%) | 288  (9.66%) |
| Pre-eclampsia + eclampsia,  *n* (%) | 5,143  (7.49%) | 1,198  (7.61%) | 3,589  (7.59%) | 1,606  (7.67%) | 1,080  (7.11%) | 336  (6.28%) | 641  (7.54%) | 205  (6.88%) |
| Gestational diabetes, *n* (%) | 1,495  (2.18%) | 474  (3.01%) | 1,041  (2.20%) | 553  (2.64%) | 127  (0.84%) | 111  (2.07%) | 62  (0.73%) | 69  (2.31%) |
| Small for gestational age,  *n* (%) | 10,552  (15.8%) | 2,706  (17.7%) | 7,354  (16.0%) | 3,608  (17.8%) | 1,544  (10.2%) | 251  (4.69%) | 862  (10.1%) | 136  (4.56%) |
| Large for gestational age,  *n* (%) | 14,620  (21.7%) | 3,063  (20.0%) | 9,880  (21.3%) | 4,375  (21.4%) | 1,733  (11.4%) | 285  (5.33%) | 1,070  (12.6%) | 179  (6.00%) |
| Spontaneous preterm birth,  *n* (%) | 6,282  (9.18%) | 1,508  (9.63%) | 4,187  (8.89%) | 2,027  (9.73%) | 2,643  (17.5%) | 688  (13.0%) | 1,509  (17.8%) | 385  (13.0%) |

^a^: analyses restricted to 1998 or later pregnancies.

**Table S3.** Sample sizes.

|  | Women | | | Male partners | | |
| --- | --- | --- | --- | --- | --- | --- |
|  | Main analyses | Only stable  couples | Both parents with  genotype data | Main analyses | Only stable  couples | Both parents with  genotype data |
| Miscarriage | 75,210 | 65,590 | 45,273 | 51,156 | 45,239 | 45,212 |
| Stillbirth | 83,900 | 74,009 | 51,396 | 55,790 | 49,794 | 49,846 |
| Hypertensive disorders of pregnancy | 83,114 | 73,339 | 50,906 | 55,273 | 49,341 | 49,377 |
| Pre-eclampsia + eclampsia | 83,114 | 73,339 | 50,906 | 55,273 | 49,341 | 49,377 |
| Gestational diabetes | 83,900 | 74,009 | 51,396 | 55,790 | 49,794 | 49,846 |
| Small for gestational age | 66,110 | 58,857 | 40,703 | 43,967 | 39,395 | 39,334 |
| Large for gestational age | 69,789 | 61,994 | 42,972 | 46,104 | 41,324 | 41,293 |
| Spontaneous preterm birth | 83,522 | 73,640 | 51,167 | 55,536 | 49,595 | 49,621 |

**SUPPLEMENTARY FIGURES**


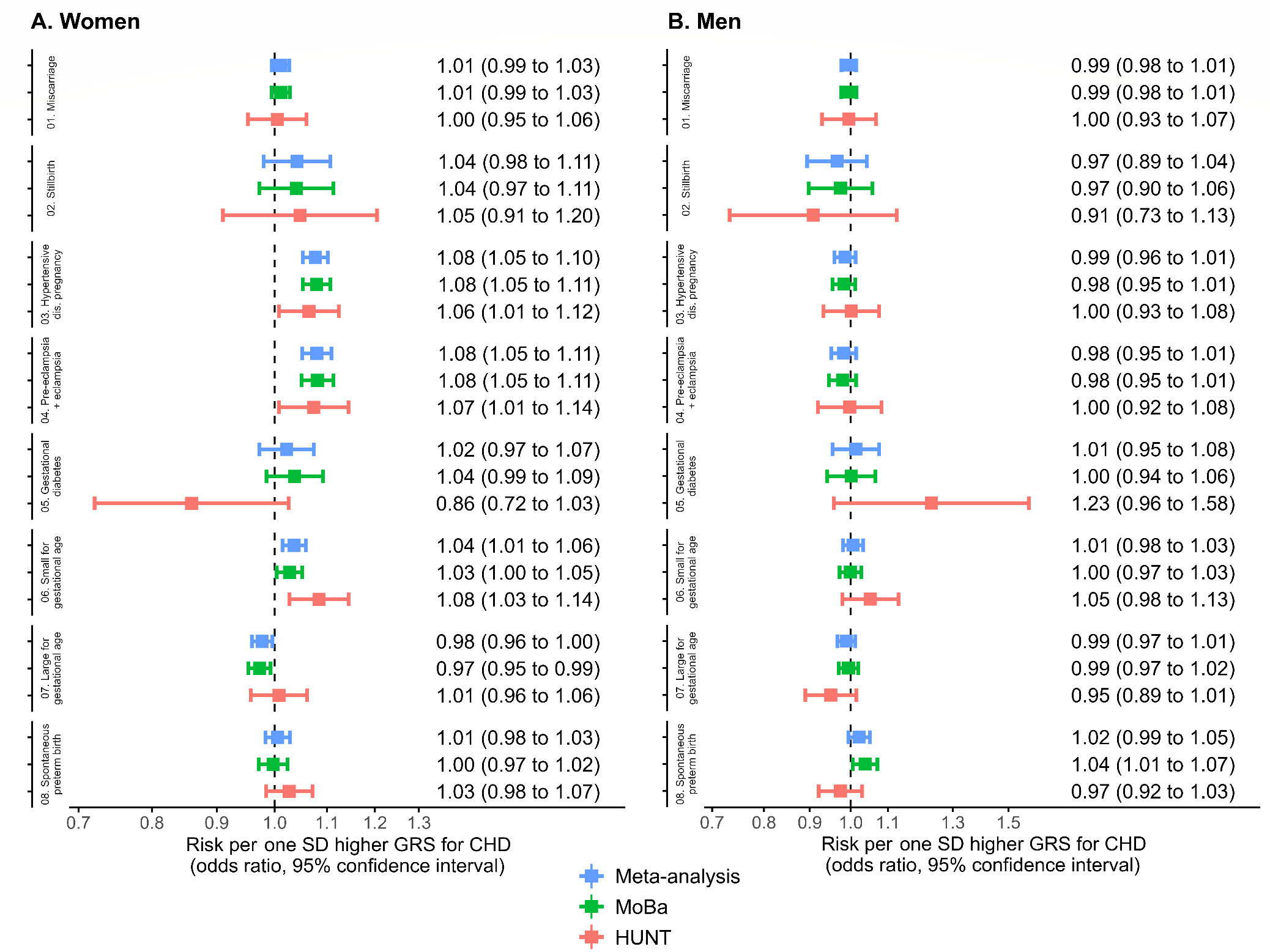
**Figure S1.** Associations between one SD higher maternal genetic risk score for coronary heart disease and adverse pregnancy outcomes (A) and between one SD higher paternal genetic risk score and adverse pregnancy outcomes in their female partners (B), in MoBa and HUNT participants individually.

Analyses are adjusted for the first 20 ancestry-informative genetic principal components and genotype batch.
